# Heatwaves and homelessness

**DOI:** 10.1177/17579139231224690

**Published:** 2024-03-05

**Authors:** J Leggat, C Dearman, S Bainbridge, E De Zoete, C Petrokofsky

**Affiliations:** Centre for Science and Policy, University of Cambridge, 10 Trumpington Street, Cambridge CB2 1QA, UK Email: jennieleggat@gmail.com; UK Health Security Agency, London, UK; Greater London Authority, London, UK; Greater London Authority, London, UK; UK Health Security Agency, London, UK

*The article highlights the deficit of evidence to understand the impact on people sleeping rough during periods of high temperature, as well as the lack of research regarding the actions that should be taken to protect them and promote their health. This dearth of evidence will become more concerning as heatwaves become more severe and more frequent due to human-induced climate change.*

Climate change is increasing the frequency, duration, and severity of extreme heatwaves across the globe.^1^ In 2022, heatwave records were broken worldwide, and 2023 has been even hotter, making the last decade the hottest on record.^2^ The impact of this on population health is clear: over 70,000 additional deaths occurred in Europe during the heatwaves of 2003,^3^ and in 2022, there were 2985 heat-related deaths in England and Wales alone.^4^ These deaths disproportionately occur in groups with pre-existing vulnerabilities,^5^ yet for individuals sleeping rough – who count among the most vulnerable and marginalised in our societies – it is not clear what interventions work to protect their health during extreme heat.

Rough sleeping or street homelessness is the act of sleeping outside or in places that are not designed for people to live in, most often due to lacking access to adequate shelter.^6^ This lack of shelter increases the exposure of people experiencing rough sleeping to adverse weather, putting them at significant risk of heat-related illness during heatwaves. For example, people experiencing rough sleeping may sit or sleep in direct sunlight or on hot surfaces such as tarmac and have limited access to air-conditioned spaces. They also tend to be concentrated in urban settings and are therefore exposed to the urban heat island effect. There are also high rates of physical and mental health conditions in the rough sleeping population that increase their vulnerability to heat-related illness. For example, it is estimated that between a quarter and half of all people experiencing rough sleeping in London were affected by physical health conditions in 2022/2023 (*personal communication: R Young, 2023, unpublished data from 2022/23 CHAIN survey*), including a significant burden of respiratory conditions that may be exacerbated due to heat-induced increases in ground-level ozone and airborne organic small particulate matter.^7,8^ Moreover, 50% of people experiencing rough sleeping have mental health needs that may be exacerbated by the heat^9^ – with a particular increase in suicide risk noted during hot weather^10^. Many of the drugs prescribed to manage these conditions (antipsychotics, antidepressants, etc.) can also inhibit the sweating mechanism and reduce cognitive alertness, increasing the risk of heat-related illness.^11^ Similarly, high rates of substance use in this population increase risk,^9^ as recreational drugs can reduce one’s ability to adapt behaviour in response to heat, and alter physiological response mechanisms.^5,11^ People experiencing rough sleeping are also three times more likely to experience social isolation than the general population,^12^ increasing vulnerability to heat-related illness not only because symptoms may be identified late, but also because this lack of social support may impair access to healthcare.^5^

**Figure:**
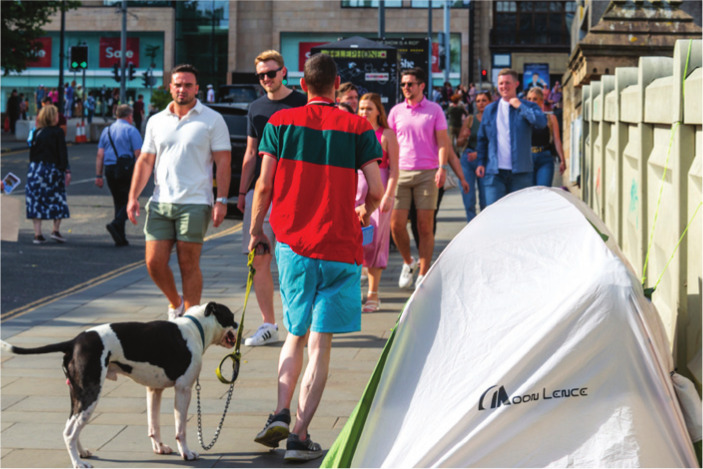


The above evidence demonstrates that people experiencing rough sleeping are at significantly higher risk of heat-related morbidity and mortality than the general population due to distinct patterns of heat exposure and pre-existing vulnerabilities. This contributes to the significantly elevated risk of hospitalisation associated with even moderately high temperatures in this population group relative to the general population.^13^ This suggests that national heat-preparedness plans should provide specific guidance on how to protect the health and wellbeing of the rough sleeping community. In recognition of this, the United Kingdom Health Security Agency (UKHSA) recently released ‘Supporting vulnerable people before and during hot weather’ guidance,^14^ that includes advice for those with responsibilities for the over 3000 people experiencing rough sleeping each night across England.^15^ In the development of this guidance, however, a lack of relevant peer-reviewed evidence regarding both the impact of adverse hot weather on those experiencing homelessness and the optimal interventions to reduce risk was identified. For example, while the guidance draws on protocols implemented to protect the rough sleeping population in cities around the globe, the efficacy and effectiveness of such protocols has not been thoroughly evaluated to our knowledge. In addition, evidence from interventions to protect the general population was used pragmatically, but such interventions must be tailored to the particular context of street homelessness – ideally being co-developed with users – in order to be most effective for the rough sleeping population. Limited evaluations have highlighted some nuanced considerations, such as the importance of allowing people experiencing rough sleeping to safely store their belongings and bring their pets into respite spaces to facilitate their use of these interventions.^16^ However, the range of factors required to ensure suitability of provision for people experiencing rough sleeping has not been established definitively, limiting the development of evidence-based guidance.

Accordingly, robust research is needed to establish: when to activate a response; how to reach and productively engage the rough sleeping population during adverse heat periods; how to identify and protect particularly vulnerable individuals; and what provisions (such as cooling centres, enhanced outreach, and overnight accommodation) are most effective for this population. We therefore urge the international research community to investigate the interplay between climate change impacts, vulnerability, and public health in this context as, without it, the health and wellbeing of those sleeping rough will be increasingly adversely impacted by heatwaves.
